# A Comparison of Second and Third Generations Combined Oral Contraceptive Pills’ Effect on Mood

**DOI:** 10.5812/ircmj.13628

**Published:** 2014-08-05

**Authors:** Mahnaz Shahnazi, Azizeh Farshbaf Khalili, Fatemeh Ranjbar Kochaksaraei, Mohammad Asghari Jafarabadi, Kamal Gaza Banoi, Jila Nahaee, Somayeh Bayati Payan

**Affiliations:** 1 Department of Midwifery, Faculty of Nursing and Midwifery, Tabriz University of Medical Sciences, Tabriz, IR Iran; 2 Department of Psychiatry, Tabriz University of Medical Sciences, Tabriz, IR Iran; 3 Department of Biochemistry, Nutrition Therapy, Community Nutrition, School of Health and Nutrition, Tabriz University of Medical Sciences, Tabriz, IR Iran

**Keywords:** Mood Disorders, Contraceptive Agents, Administration, Oral

## Abstract

**Background:**

Most women taking combined oral contraceptives (COCs) are satisfied with their contraceptive method. However, one of the most common reasons reported for discontinuation of combined oral contraceptives (COCs) is mood deterioration.

**Objectives:**

This study aimed to compare effects of the second and third generation oral contraceptive pills on the mood of reproductive women.

**Materials and Methods:**

This randomized, double-blind, controlled clinical trial was conducted in reproductive women at health centers in Tehran, Iran. Participants were randomized into the second and third generation oral contraceptive groups. Positive and negative moods were recorded using positive affect, negative affect scale (PANAS) tools at the end the second and fourth months of the study. Data analysis was carried out using ANOVA and P Values < 0.05 was considered significant.

**Results:**

Statistically significant difference was seen in positive and negative mood changes in women receiving contraceptive pills. The second generation oral contraceptive pills resulted in a decrease in positive mood (95% CI: 43.39 to 38.32 in second month and 43.39 to 26.05 in four month) and increase in negative mood (95% CI: 14.23 to 22.04 in second month and 14.23 to 32.26 in four month - P < 0.001), but the third generation led to an increase in positive mood (95% CI: 22.42 to 25.60 in second month and 22.42 to 33.87 in four month) and decrease in negative mood (95% CI: 36.78 to 31.97 in second month and 36.78 to 22.65 in four month - P < 0.001).

**Conclusions:**

Third generation combined oral contraceptive pills have a better effect on mood in women in reproductive ages than the second generation pills. It can be recommended as a proper combined oral contraceptive in Iran.

## 1. Background

Most women taking combined oral contraceptives (COCs) are satisfied with their contraceptive method (1). However, mood deterioration is one of the most common reasons reported for discontinuation of combined oral contraceptives (COCs) (2). More than 30% of women using COCs discontinue or change their medication due to adverse mood effects (3). The discontinuation rates among new pill starters are reported to be about 32% to 33% within six months of treatment, and most of those who discontinue or switch COCs do so within the first three months of treatment (3, 4).

Birth control pills are a class of drugs, widely studied since 1960 and used by more than 70 million women daily (5). In a national research in the U.S. on the contraceptive methods, it was concluded that the oral contraceptives had the highest rate of use, and oral contraceptives in 15 to 44-year-old women was the first grade selected method (18.9%) (6). In the current circumstances of Iran, oral contraceptives are one of the most practical and effective methods for preventing pregnancy, which are chosen by many couples (7). The oral contraceptive pills are a combination of two components of estrogen and progestin, and they are divided into three generations based on their type of progesterone. The first generation progestin includes norethindrone, lynestrenol, ethynodiol diacetate, and norethisterone. The second generation includes levonorgestrel, and norgestrel. And the third generation includes desogestrel, gestodene, and norgestodene. Due to their causality with breast cancer, first-generation progestins are not used and have more side effects than the second and third generation ones (8).

Augmented levels of androgens have been related to clinical mood disorders in women. For example, increased free testosterone (FT), the bioactive form of testosterone, was observed in women with premenstrual syndrome (PMS) (9) and in depressed women (10, 11). In contrast, healthy women without PMS were found to have decreased free testosterone levels premenstrually (12).

To improve tolerability (and safety) of COCs, new progestogens have been developed over the years. Indeed, COCs with anti-androgenic progestogens such as drospirenone and desogestrel appear more favorable in terms of mood symptoms than progestogens with a more androgenic profile such as levonorgestrel (13).

The most important reason for discontinuation of taking contraceptive pills is fear of side effects, (3) and mood change has been referred to as one of the most usual reasons for discontinuance of these pills (14, 15). Despite the significant number of studies focusing on side effects of contraceptive pills, still, mood change is one of the contentious side effects of the pills (7). Considering the suggestion of the published review in the United States for further research in order to contribute to the literature with regard to the relationship between taking the pill and mood (16), and considering the fact that the second generation pills (LD) are the most consumed oral contraceptive in Iran, and also for the reason that the percentage of using this method has increased from 18.4% in 2000 to 19.3% in 2005 (17).

## 2. Objectives

The present study aimed to investigate the effects of the second and third generation oral contraceptives on mood in women in reproductive ages.

## 3. Materials and Methods

### 3.1. Design

This was a randomized clinical trial study conducted on married women at reproductive age (15-45 years old) who were referred to the health centers in east region of Tehran, Iran for oral contraceptive method, in 2011 and 2012. A total of 82 women were randomly allocated into group receiving second generation contraceptive pills (LD^™^: low-dose estrogen) or the group receiving third generation pills (Marvelon^®^) based on a block randomization with block sizes of 4 and 6 and an allocation ratio of 1: 1. The allocation sequence was identified by computerized random numbers. Sequentially numbered sealed envelopes with the same shape and size containing LD or Marvelon tablets were used to conceal the allocation and to maintain the blinding. Every envelope contained 21 pills of LD or Marvelon. The participants were instructed to take one tablet per day for 21 days.

The envelopes were prepared by a person who was not involved in the recruitment, data collection, or data analysis. Therefore, the researchers and participants were unaware of the kind of tablets given to each participant (blinding). The LD tablets, contained 0.15 mg levonorgestrel and 0.03 mg ethinyl estradiol, produced by Aboureihan Company in Iran, and Marvelon^®^ pills contained 0.15 mg desogestrel and 0.03 ethinyl estradiol made by Iran Hormone in Iran. Marvelon tablets were the same as the LD tablets in shape, color, and size, and were thus indistinguishable from the LD tablets.

### 3.2. Setting and Participants

Women who met the following criteria were eligible for recruitment: 1. age of 15 to 45 years, 2. no absolute or relative contraindication for taking compound contraceptive pills, 3. no affective illnesses on mood based on the patient and the physician’s examination of the center, 4. no experience of critical events in the last three months, 5. no consumption of hormonal contraceptive methods in the whole last year, 6. no consumption of psychoactive drugs and 7. body mass index (BMI) ≥ 18 and ≤ 29.

The subjects were selected from women referred to two public health care centers affiliated to Shahid Beheshti University of Medical Sciences, Tehran, Iran. These centers had the highest numbers of clients for family planning services among the centers affiliated to the university. All participants had household records at the centers for receipt of primary health care, including family planning services in previous years. To contact the participants, we found their phone numbers from the records. Some of the eligibility criteria were checked over the phone. The potentially eligible subjects were invited to attend the centers. At the centers, the women were further informed about the study, the eligibility criteria were checked precisely, and informed consent forms were signed by the eligible women.

The research protocol was approved (Code 9087) by the Ethics Committee of Tabriz University of Medical Sciences and registered at the Iranian Registry of Clinical Trials with IRCT201107186709N4. The sample size was calculated using STATA software version 9.2 (Statasoft Inc, USA) based on a study by Maleki et al. (18). Considering significance level of 0.05, a power of 0.80, and a 10% probable drop in the sample, the required sample size was calculated to be 41 participants per group.

### 3.3. Data Collection

Data collection was carried out by the use of socio-demographic and reproductive history questionnaire and mood measurement questionnaire (PANAS) (19). The mood questionnaire consisted of 20 items in order to measure positive and negative mood. Ten items for positive mood included: enthusiast, excitement, power, motivation, self-esteem, awareness, high morality, being determined concentration, attentiveness, and being active; and ten items for negative mood included: stress, disappointment, guilt, fear, hostility, being offensive, sensitive, feeling ashamed and shyness, angriness and anxiety. The questionnaire measured individuals’ mood with regard to every item in the last week. Numbers ranged from 1 to 5 in Likert scale and answers were categorized as (1) never, (2) little, (3) average, (4) high, and (5) very high.

Scores ranged from 10 to 50 per positive and negative mood. The questionnaire investigated mood change per week. The PANAS is standard and benefits from international credibility and considering its validity in determining mood and its further validity was unnecessary. Mohammadi Yeghaneh (20) translated and use the Persian version of this questionnaire (Alpha chronbach coefficient: 0.87).

The questionnaire was completed (before intervention by the researcher and co-researcher) by interviewing the researched participants. The participants were invited to complete the mood questionnaire on weekly basis up to one month prior to intervention. Also, the participants were invited to fill out the questionnaire during this month (before intervention) using the previous method. After the group allocation, the researcher and the study subjects were unaware of the types of pills given to them. After giving the envelopes containing 21 pills, they were instructed on how to use the pills, which was once a day for 21 days with 7-day interval to start the next cycle of pills. At the end of the second and fourth month of using the pills, the subjects were asked to complete the PANAS questionnaire by phone calls. The duration of the intervention took four months, and data collection was done during three stages (pre-intervention, and 2 and 4 months intervention).

### 3.4. Statistical Analysis

Data for qualitative variables were reported as frequency (percentage) and quantitative variables as mean difference and standard deviation (SD). Kolmogorov Smirnov test was used to assess the data distribution normality. To compare the qualitative variables in the two groups, χ^2^ (chi-square) was used with precise P Value. To compare the mood variable in the groups, independent samples t-test and analysis of covariance (ANCOVA) was used respectively to compare baseline and measurement in post-intervention phase. In this analysis, the baseline measurement of mood variable and confounding variables (age) were adjusted. In addition, for checking the time measurement changes in each of the two groups, analysis of variance (ANOVA) with repeated measures and Holm-Sidak post-hoc tests were used. Data analysis was done through SPSS for Windows 17.0 (SPSS Inc. Chicago, IL, USA) and P Value < 0.05 was set as a significant level.

## 4. Results

**Figure 1.**
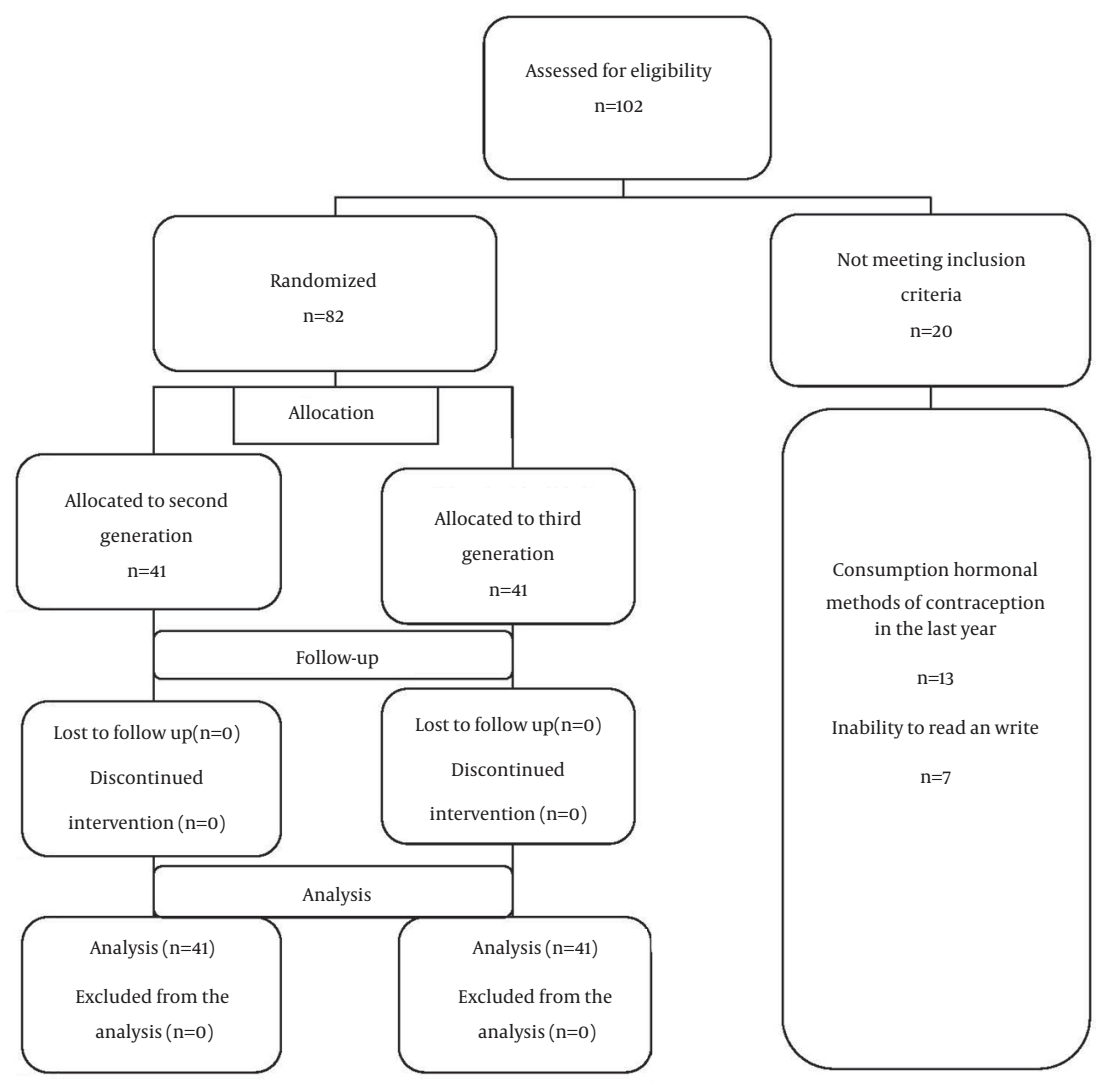
Study Protocol

In this study, 102 women in their reproductive age who wished to participate in the study were selected. Twenty women were excluded from the study because they did not meet the inclusion criteria. Therefore, the study was conducted on 82 women in their reproductive age in two groups of 41; one group received second-generation oral contraceptives (LD^™^) and the second group received third-generation oral contraceptives (Marvelon^®^) (Figure 1). There was no loss to follow-up and all participants continued the study to the end. Both groups were similar in terms of the personal and social characteristics, the average age in the group receiving second generation and third generation was 29 ± 7.01 and 28 ± 7.93 years, respectively. The majority (34.1%) of patients in both groups had secondary school education and the majority of patients in the group receiving second generation pills (34.1%) were housewives and in the group receiving third generation (34.1%) worked at home, other personal and social characteristics are shown in (Table 1).

The results of the ANOVA with adjustment for baseline values showed that in months 2 and 4, the mean score of positive mood and negative mood had a statistically significant difference between the two groups in month 2 and month 4, with Considering mean score of positive and negative mood with controlling the effect of mean score before study (P < 0.001). Mean score of positive mood in the second and four month in the group using third generation pills was higher compared to the second generation, and mean score of negative mood in the second and four month in the group using second generation pills was higher compared to the third generation. Furthermore, positive and negative mood changes in the fourth month had significant increase in the third and second generation oral contraceptive (Table 2).

**Figure 2.**
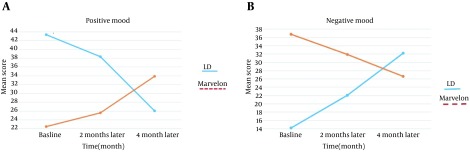
Mean Negative and Positive Mood in the Two Groups Receiving Second and Third Generation Contraceptive Pills

Inter-group comparison using ANOVA with repeated measures revealed that in both groups, the second and third generation, and mean change in the positive and negative mood score in the second and fourth month after intervention were statistically significant compared to the pre-intervention phase (P < 0.001). Positive mood scores with the second generation pills at the end of the second and fourth months decreased, while negative mood scores increased. Positive mood scores in the third generation group after the second and fourth months increased, while negative mood scores had decreased (Figure 2) (Table 3).

**Table 1.** Demographic Characteristics of the Study Participants in the Group Receiving Second Generation (LD) and Group Receiving Third Generation (Marvelon)

Variables	Group Receiving LD™, No. (%)	Group Receiving Marvelon^®, ^No. (%)	Statistical Indicators
**Age, y**			t = 0.87; df = 80; P = 0.38
Less than 20	4 (9.75)	8 (19.51)	
21-30	24 (58.53)	18 (43.90)	
31-40	9 (21.95)	11 (26.82)	
Over 40	4 (9.75)	4 (9.75)	
Mean ± SD	29.29 ± 7.01	27.85 ± 7.93	
**Education level**			χ^2^ = 0.001; df = 3; P = 1
Primary	10 (24.4)	10 (24.4)	
Secondary	14 (34.1)	14 (34.1)	
High school	13 (31.7)	13 (31.7)	
University	4 (9.8)	4 (9.8)	
**Employment **			χ^2^ = 1.07; df = 3; P = 0.81
Housewife	14 (34.1)	10 (24.4)	
Work outside the home	12 (29.3)	13 (31.7)	
Work at home	11 (26.8)	14 (34.1)	
Student	4 (9.8)	4 (9.8)	
**Children**			χ^2^ = 0.05; df = 1; P = 0.82
Yes	16 (39.0)	18 (43.9)	
No	20 (61.0)	23 (56.1)	

**Table 2.** Mean Score of Positive and Negative Mood at Follow-Up the Two Groups Receiving Second and Third Generation Contraceptive Pills^a,b,c^

Mood	Group Receiving Second Generation, Mean ± SD	Group Receiving Third Generation, Mean ± SD	Mean Difference (95% CI)	Statistical Indicators
**Positive mood**				
Pre-intervention	43.39 ± 2.62	22.42 ± 8.74	20.95 (18.11, 23.78)	t = 14.68, df = 80, P < 0.001
Two months later	38.32 ± 5.58	25.60 ± 8.36	-6.42 (-9.80, -3.03)	F = 14.25^b^, df = 1, P < 0.001
Four months later	26.05 ± 6.27	33.87 ± 9.38	-20.75 (-26.63, -14.87)	F = 49.43^b^, df = 1, P < 0.001
**Negative mood**				
Pre-intervention	14.23 ± 3.88	36.78 ± 4.37	-22.55 (-24.370, -20.730)	t = 24.68, df = 80, P < 0.001
Two months later	22.04 ± 6.26	31.97 ± 5.76	7.95 (14.63, 1.27)	F = 5.61 ^b^, df = 1, P = 0.02
Four months later	32.26 ± 6.24	26.65 ± 6.86	12.14 (3.60, 20.64)	F = 8.08 ^b^, df = 1, P < 0.001

^a^ Abbreviations: CI, mean difference (confidence interval).

^b^ Minimum and maximum score of Positive and negative mood was 10-50.

^c^ ANOVA.

**Table 3.** Mean Changes in Positive and Negative Mood According to Follow-Up of the to the Two Groups Receiving Second and Third Generation Contraceptive Pills ^a,b^

MOOD	Group Receiving Second Generation, Mean ± SD	Group Receiving Third Generation, Mean ± SD	Mean Difference (95% CI)	Statistical Indicators
**Positive mood**				
Mean Difference Two Months Later and Pre-Intervention	-5.06 ± 4.77	3.16 ± 3.01	-8.22 (-9.98, -6.47)	t = -9.31, df = 80, P < 0.001
Mean Difference Four Months Later and Pre-Intervention	-17.33 ± 6.69	11.43 ± 7.89	-28.77 (-31.99, 25.55)	t = -17.79, df = 80, P < 0.001
Repeated Measures ANCOVA (Inter-group)	F = 196.90, df = 2, P < 0.001	F = 65.01, df = 2, P < 0.001		
**Negative mood**				
Mean Difference Two Months Later and Pre-Intervention	7.81 ± 5.62	-4.80 ± 4.66	12.61 (-14.89, 10.340)	t = -11.05, df = 80, P < 0.001
Mean Difference Four Months Later and Pre-Intervention	18.03 ± 7.46	-10.12 ± 6.67	28.15 (31.24, 25.04)	t = 17.99, df = 80, P < 0.001
Repeated Measures ANCOVA(Inter-group)	F = 144.24, df = 2, P < 0.00	F = 65.02, df = 2, P < 0.001		

^a^ Abbreviation: Mean Difference (Confidence Interval).

^b^ T-test.

## 5. Discussion

The results from this study show that COC containing 0.15 mg desogestrel/0.03 mg ethinyl estradiol (EE) significantly improved positive and negative mood symptoms, compared with COC containing 0.15 mg Ievonorgestrel/0.03 mg ethinyl estradiol (EE) and third generation OCPs had a beneficial effect on positive and negative mood symptoms compared second generation OCPs.

Positive mood score in the second month decreased about 11.7% in second generation COCs and increased about 18.3% in third generation COCs and in the fourth month decreased about 40.0% in second generation COCs and increased about 51.1% in third generation COCs.

Negative mood score in the second month increased approximately 54.9% in second generation COCs and decreased about 13.7% in third generation COCs and in fourth month increased 55.88% in the second generation and decreased about 27.54% in third generation COCs.

However, there are divergent results from studies regarding the effect of COCs on mood, with some showing improvement; some with deterioration and others demonstrated no effect. Mood symptoms can be irritability and depression (20) but can also be symptoms that occur with the use of COCs, depending on the profile of the progestogens and the dose and type of estrogen (21).

The results of this study are consistent with the results of some previous studies. In the study by Nyberg (22) with the aim of improving the physical and mood symptoms in women using third generation OCPs, results showed that the level of negative mood reduced and positive mood increased 3 months after taking 250 mg norgestimat and 35 mg ethinyl estradiol. Another study reported that in women taking second generation of oral contraceptives, mood swings, depression and fatigue was higher than the control group receiving placebo (23) and other studies have also reported that the level of negative mood increased in second generation oral contraceptives consumers (24-26).

The present results appear inconsistent with the results of some other studies, Jarva and Oinonen (27) showed that among three groups of women with first, long term and non-previously consumers of third generation OCPs, positive emotional response has been reduced in the current consumers. The difference in these two studies may be due to different in sample size and characteristics of the study participants. In the study of 107 student in Canada (40 women using 3rd generation OCPs, 36 women who were not OCP consumer and 31 men) and mean age of participants was 19 years. In the present study the ages ranged between 15 to 45 years.

The double-blinding and no loss to follow-up were important strengths of this study. The duration of the treatment in this study was relatively short (4 months) and we did not have any follow-up after oral contraception use because of time and financial limitations. Although the positive and negative mood score is a subjective outcome (self-reporting), the results may not be affected by subjectivity because of the double blinding of the study. Other outcomes such as vomiting and menstrual bleeding symptoms, androgen hormone serum levels example testestron free, testestron total and DHEA-S and spotting were not assessed, and the safety was only assessed by self-reporting because of financial limitations. It is also recommended that health care staff provide accurate information about possible changes in mood change for people applying oral contraceptive methods, and according to each individual’s condition recommend the best oral contraceptive.

The most common type of oral contraceptive used in Iran is the second generation oral contraceptive LD^™^, which is freely distributed in health centers. Therefore, it is necessary for women who request to use these contraceptive methods to be educated and counseled properly using them. The third generation contraceptive pills can be recommended to women who request to use oral contraceptives.
